# Predictive effects of the interactions among cesarean section, birth weight, and preterm birth on the risk of atopic dermatitis in children

**DOI:** 10.1002/kjm2.12826

**Published:** 2024-04-17

**Authors:** Chih‐Kai Wong, Cheng‐Fang Yen, Yi‐Lung Chen

**Affiliations:** ^1^ Department of Dermatology MacKay Memorial Hospital Taipei Taiwan; ^2^ Department of Psychiatry Kaohsiung Medical University Hospital, Kaohsiung Medical University Kaohsiung Taiwan; ^3^ Department of Psychiatry School of Medicine, College of Medicine, Kaohsiung Medical University Kaohsiung Taiwan; ^4^ Department of Psychology Asia University Taichung Taiwan; ^5^ Department of Healthcare Administration Asia University Taichung Taiwan

Dear Editor,

Cesarean section (CS) and level of maturity at birth, as indicated by birth weight and gestational age, may influence the incidence of atopic dermatitis (AD); however, the findings of studies have been inconsistent. For example, research has revealed that CS increased the risk of AD by altering neonatal gut microflora colonization,^1^ whereas a meta‐analysis did not observe a significant association between CS and an increased risk of AD.^2^ Not accounting for the interaction effects of CS, preterm birth, and birth weight when assessing the risk of AD may partially explain the inconsistent findings of other studies.^3^

This population‐based cohort study used the data of 1,884,701 children born between 2004 and 2017 from the Taiwan Maternal and Child Health Database to investigate the interaction effects of CS, preterm birth (gestational age shorter than 37 weeks), and birth weight (low and high classified by <2 and >2 standard deviations of the mean birth weight for gestational age, respectively) on AD. In total, 52.1% were boys, 35.9% were delivered by CS, 9.0% were preterm, 1.8% had a low birth weight, and 3.2% had a high birth weight. Children were identified as having AD in accordance with the *International Classification of Diseases (ICD), Ninth* (ICD‐9 code: 691) *and Tenth Revisions* (ICD‐10 code: L20) if they had more than two outpatient diagnoses or one inpatient diagnosis between January 1, 2004, and December 31, 2018. The Cox proportional hazards regression model was used to analyze the associations of the interaction between CS, preterm birth, and birth weight with the risk of AD.

In total, 26.2% of children had the diagnosis of AD during the follow‐up period. The interaction of CS and preterm birth (adjusted hazard ratio [aHR] = 1.04, 95% CI: 1.02–1.06) and the interaction of CS and high birth weight (aHR = 1.07, 95% CI: 1.03–1.11) were significantly associated with an increased risk of AD (Table 1). Conversely, the interaction of preterm birth and high birth weight was significantly associated with a decreased risk of AD (aHR = 0.90, 95% CI: 0.81–1.01).

**TABLE 1:** The associations between the interactions of cesarean section, preterm birth, and birth weight and atopic dermatitis.

	Atopic dermatitis	
	aHR (95% CI)	*p*
Cesarean section	1.05 (1.04–1.06)	<0.0001
Preterm birth	0.95 (0.93–0.96)	<0.0001
Birth weight 1 (low vs. normal)	0.95 (0.92–0.98)	0.0006
Birth weight 2 (high vs. normal)	0.80 (0.79–0.83)	<0.0001
Cesarean section × preterm birth	1.04 (1.02–1.06)	0.0004
Cesarean section × birth weight 1	1.02 (0.98–1.07)	0.3192
Cesarean section × birth weight 2	1.07 (1.03–1.11)	<0.0001
Preterm birth × birth weight 1	0.93 (0.79–1.11)	0.3997
Preterm birth × birth weight 2	0.90 (0.81–1.00)	0.0422
Preterm birth × cesarean section × birth weight 1	1.16 (0.96–1.4)	0.1209
Preterm birth × cesarean section × birth weight 2	1.09 (0.96–1.23)	0.1969

*Note*: Adjusted hazard ratio was conducting with controlling for controlling for maternal age of childbirth, mother's atopic dermatitis, and child's sex.

Abbreviation: aHR, adjusted hazard ratio.

CS, preterm birth, and high birth weight may increase the risk of AD through a range of underlying mechanisms. CS may alter neonatal gut microflora colonization.^1^ Preterm birth may accelerate transepidermal water loss in infants.^4^ High birth weight is associated with increased concentrations of total serum immunoglobulin E.^5^ The interactions of these underlying physiological mechanisms may further increase the risk of AD. However, the present study also observed that the interaction of preterm birth and high birth weight was significantly associated with a decreased risk of AD.

The present study did not adjust the effects of several factors such as antibiotic use in early life for preterm birth and family history of allergies on the associations of the interactions among CS, birth weight, and preterm birth with AD. Moreover, the proportions of low (2.1%), normal (92.8%), and high birth weight infants (5.0%) in this study were uneven and might influence the relationship between birth weight and the risk of AD. The etiology of the interacting effects among CS, preterm birth, and high birth weight warrants further investigation. The findings of this study demonstrate the value of monitoring the risk of AD in children delivered by CS with preterm birth or high birth weight.

## CONFLICT OF INTEREST STATEMENT

The authors have no conflicts of interest to disclose.

## ETHICS STATEMENT

This study was approved by the Research Ethics Committee of China Medical University Hospital (approval number: CMUH108‐REC1‐142). Participant consent was waived because that this study derived anonymous data form massive data base.
